# Pressure-Induced Phase Transformations of Quasi-2D Sr_3_Hf_2_O_7_

**DOI:** 10.1021/acs.jpcc.3c01596

**Published:** 2023-08-01

**Authors:** M. C. B. Barbosa, E. Lora da Silva, P. Neenu Lekshmi, M. L. Marcondes, L. V. C. Assali, H. M. Petrilli, A. M. L. Lopes, J. P. Araújo

**Affiliations:** † IFIMUP, Institute of Physics for Advanced Materials, Nanotechnology and Photonics, Department of Physics and Astronomy, Faculty of Sciences, University of Porto, Rua do Campo Alegre, 687, 4169-007 Porto, Portugal; ‡ High Performance Computing Chair, University of Évora, Largo dos Colegiais 2, 7004-516 Évora, Portugal; § Instituto de Física, Universidade de São Paulo, Rua do Matao 1371, 05508-090 São Paulo, SP, Brazil

## Abstract

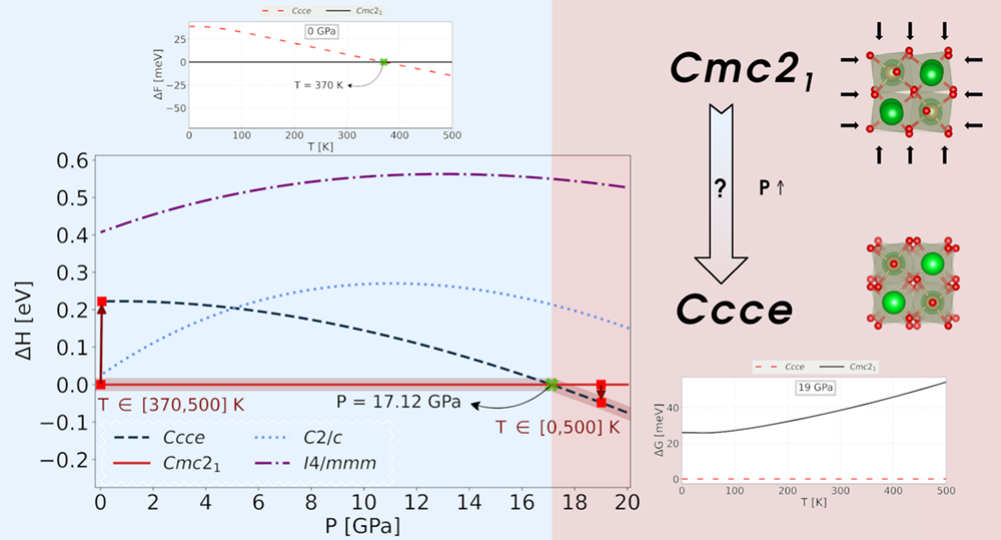

We present an abinitio study of the quasi-2D layered perovskite Sr_3_Hf_2_O_7_ compound, performed within the framework of the density functional theory and lattice dynamics analysis. At high temperatures, this compound takes a *I4/mmm* centrosymmetric structure (S.G. n. 139); as the temperature is lowered, the symmetry is broken into other intermediate polymorphs before reaching the ground-state structure, which is the *Cmc*2_1_ ferroelectric phase (S.G. n. 36). One of these intermediate polymorphs is the *Ccce* structural phase (S.G. n. 68). Additionally, we have probed the *C2/c* system (S.G n. 15), which was obtained by following the atomic displacements corresponding to the eigenvectors of the imaginary frequency mode localized at the Γ-point of the *Ccce* phase. By observing the enthalpies at low pressures, we found that the *Cmc*2_1_ phase is thermodynamically the most stable. Our results show that the *I4/mmm* and *C2/c* phases never stabilize in the 0–20 GPa range of pressure values. On the other hand, the *Ccce* phase becomes energetically more stable at around 17 GPa, surpassing the *Cmc*2_1_ structure. By considering the effect of entropy and the constant-volume free energies, we observe that the *Cmc*2_1_ polymorph is energetically the most stable phase at low temperature; however, at 350 K, the *Ccce* system becomes the most stable. By probing the volume-dependent free energies at 19 GPa, we see that *Ccce* is always the most stable phase between the two structures and also throughout the studied temperature range. When analyzing the phonon dispersion frequencies, we conclude that the *Ccce* system becomes dynamically stable only around 19–20 GPa and that the *Cmc*2_1_ phase is metastable up to 30 GPa.

## 1 Introduction

Rational design and the discovery of new materials are essential toward the advancement of future technological innovations. Perovskite structures (with general chemical formula ABX_3_, where A and B are typically large and small cations, respectively, and X is an anion) are one of the most versatile classes of materials, with a variety of fascinating characteristics such as magnetism, superconductivity, thermoelectricity, electrocatalysis, ferroelectricity, and optical properties.^1,2^ While the greater part of the known perovskite oxides are centrosymmetric, a small fraction of these crystallizes as non-centrosymmetric (polar perovskites).^3^ The polar phase enables the emergence of ferroelectricity, piezoelectricity, and non-linear optical effects which are crucial to modern electronic devices, such as sensors, actuators, tunnel junctions for non-volatile memory, transducers for medical imaging, and solar cells.^1,2,4−6^

Among perovskites, naturally layered perovskite oxides, such as quasi-2D Ruddlesden–Popper (RP) and Dion–Jacobson (DJ) structures, have particular interest because of their recently discovered mechanism for ferroelectricity, termed as “hybrid improper ferroelectricity” (HIF).^7,8^ Structures with HIF exhibit two non-polar structural distortion modes, which lead to the third polar distortion mode, and are known as the trilinear coupling, resulting in a macroscopic spontaneous polarization.^7,9^ For perovskite structures which undergo distortions toward the *Pnma* space group (i.e., double-perovskite structures), the HIF phenomenon leads to an antiferro-distortive displacement of the *A*-site cations and the adjacent layer displacement in opposite directions.^10,11^ The displacements of the adjacent layers cancel each other for ABO_3_ single perovskites, leading to non-polar phases. However, naturally layered quasi-2D perovskites, with disconnected octahedral layers and having two inequivalent crystallographic A-sites, exhibit macroscopic layer polarization,^12,13^ which are oriented oppositely with slightly different intensities. In the RP structured naturally layered oxides, with the (AO)(ABO_3_)_*n*_ stoichiometry form, the *n*-layers of the ABO_3_ perovskite slabs are stacked between the AO rock-salt layers, with a relative shift of the neighboring perovskite slabs by a (,) translation. By increasing the value of *n* from 1 to *∞*, it is possible to tune the structural dimensionality of these perovskites from quasi-2D to 3D. At high temperatures, each of the *n* layers consists of vertex sharing oxygen octahedra with a B-ion sitting at its center.^7,9^ As temperature decreases, these systems can undergo structural phase transitions due to reorientation of oxygen octahedra, leading to HIF. The HIF is also predicted to occur in oxide superlattices.^14^ In addition to HIF, the octahedral reorientations in layered perovskites are of continuing interest because these effects also trigger many interesting electronic properties, such as high-*T*_c_ superconductivity, colossal magnetoresistance, metal-insulator transitions, and magnetic ordering.^15^

Recent theoretical works from Liu et al.^16^ and da Silva et al.^17^ confirmed that Sr_3_Hf_2_O_7_ (SHO), a *n* = 2 quasi-2D RP layered perovskite system, with a ferroelectric ground state *Cmc*2_1_ (S.G. n. 36), is similar to the previously reported HIF systems, such as Ca_2_Mn_3_O_7_ and Ca_2_Ti_3_O_7_, in ref (15). SHO is also characterized by a high-temperature *I4/mmm* (S.G. n. 139) centrosymmetric paraelectric structure. Previous studies showed that by applying group theoretical analysis, it was possible to probe potential intermediate polymorphs that form as the temperature is lowered, enabling a transition pathway as *I4/mmm* → *Cmcm* (S.G. n. 63) → *Cmca* (S.G. n. 64) → *Cmc*2_1_ (S. G. n. 36).^7,17^ The *Ccce* (S.G. n. 68) structural phase is a possible polymorph; however, it is not evidenced as being an intermediate structure, occurring for these structural RP symmetries, according to group theoretical analysis—a condensation of the X_1_^–^ zone boundary mode would have to occur upon symmetry breaking of the centrosymmetric system. It was also found by computing the phonon dispersion curves of the *Ccce* phase^17^ that the system is dynamically unstable at 0 K and 0 GPa, evidencing negative phonon modes localized at two of the high-symmetry points of the Brillouin zone (BZ): Γ- and **Y**-points. Therefore, the *Ccce* polymorph does not have the potential to crystallize at room conditions. We must note that such a phase has been experimentally evidenced as an intermediate phase for increasing temperatures in systems with similar stoichiometry, i.e., Ca_3_(Mn/Ti)_2_O_7_,^18,19^ thus making it plausible for this polymorph to form through the first-order transition for the SHO system as well.

Only recently have high-pressure (HP) studies been devoted to understand the properties of the perovskite systems and the respective transition pathways. Pressure is an important thermodynamic variable, for it enables a precise control over the interatomic distances and hence the atomic interactions, in turn driving phase-transitions. Almost all materials will undergo several phase-transitions under compression, thus generating new polymorphs with promising properties, different to those properties of materials under room conditions. It has been found that factors which are known to control the ABO_3_ perovskites pressure response, such as the A- and B-site cation formal charges, the tolerance factor, and the B-site chemical environments, also affect the pressure response of the layered perovskites.^20^ Therefore, the interest in applying pressure to probe the structural, electronic, and vibrational properties to such structures.

A recent experimental study on Sr_3_Sn_2_O_7_ compound evidences a sequence of pressure-induced phase transitions as *Cmc*2_1_ → *Pbcn* (S.G. n. 60) → *Ccce* → *I4/mmm* at room temperature.^21^ Such transition pathway would match the sequence of temperature-dependent structural transitions observed for this compound between 77 and 1000 K. For the Ca_3_M_2_O_7_-based oxides (M = Ti, Mn),^22^ ferroelectric switching is inhibited by the irregular domains induced by the intermediate *Ccce* phase, and the switching may be realized when the intermediate phase is suppressed by applying chemical pressure. Moreover, it has been evidenced that the O octahedra tilt and rotation modes of Ca_3_Ti_2_O_7_ undergo softening when hydrostatic pressure or heating is considered.^23,24^ This behavior under temperature and pressure reveals the softness of the antiphase tilt, which indicates the importance of the partially occupied *d*-orbitals of the transition metal ions, since these determine the stability of the oxygen octahedra distortion.^23,24^ Moreover, through Raman measurements, it was observed that under isotropic pressure, the polyhedra tilts of the Ca_3_Mn_2_O_7_ compound can be suppressed within the low-pressure regime (1.4–2.3 GPa).^25^ On the other hand, through DFT calculations (0 K) performed on the Ca_3_Ti_2_O_7_ compound, it was recently found by computing the enthalpies for several pressure values, up to 20 GPa, that in fact the *Ccce* phase can become energetically more favorable than *Cmc*2_1_, with the transition occurring slightly below 15 GPa.^20^

The goal of this work is to inspect, through first-principles calculations, the evolution of the structural properties and the energetic and dynamic stabilities of the *Cmc*2_1_ and *Ccce* structural phases of the SHO compound for increasing values of applied hydrostatic pressure. Furthermore, enthalpy results on the *I4/mmm* and *C2/c* structural phases have also been computed.

To probe the energetic (thermodynamic) stability, we evaluate the enthalpy difference curves of the *Ccce*, *C2/c*, and *I4/mmm* structural phases, relative to the ground-state *Cmc*2_1_ phase at 0 GPa, for hydrostatic pressures up to 20 GPa. We also analyze the free energies, where the zero-point energy is considered, in order to infer the energetic stability trend between the *Ccce* and *Cmc*2_1_structures. It is worth mentioning that we have considered not only the harmonic approximation (HA) but also the quasi-HA (QHA) at constant pressure of 19 GPa. Upon these results, the dynamic stability was probed by analyzing the phonon dispersion curves of the two energetically lowest structural phases—*Ccce* and *Cmc*2_1_—at relevant pressure values.

## 2 Theoretical Methodology

Density functional theory (DFT)^26,27^ calculations were performed through the use of the Quantum Espresso (QE) code.^28−30^ For the exchange-correlation (xc) functional, the semi-local generalized-gradient approximation with the Perdew—Burke–Ernzerhof revised for solids (PBEsol)^31,32^ was used.

First, variable-cell relaxation calculations were performed for each structural phase (*Cmc*2_1_, *Ccce*, *C2/c*, and *I4/mmm*) of the SHO compound and for each applied hydrostatic pressure value, ranging from 0 GPa up to 20 GPa. In these structural relaxations, both the atomic positions and the unit-cell parameters were allowed to change by constraining the system to the targeted pressure value. The projector augmented wave (PAW)^33^ data sets were used to treat semi-core electronic states, with the Sr[4*s*^2^ 4*p*^6^ 5*s*^2^], Hf[5*s*^2^ 5*p*^6^ 5*d*^2^ 6*s*^2^], and O[2*s*^2^ 2*p*^4^] electrons being included in the valence shell. Additionally, the variable cell-shape relaxation, considering the damped Beeman ionic dynamics and the Wentzcovitch extended Lagrangian for the cell dynamics,^34^ was performed with a plane-wave kinetic-cutoff of 70 Ry. The electronic BZ was sampled with a Γ-centered Monkhorst–Pack mesh^35^ and defined with 6 × 6 × 12 subdivisions for the *Ccce* and *C2/c* phases; 12 × 12 × 6 subdivisions for the ground-state *Cmc*2_1_ system, and 14 × 14 × 14 subdivisions for the *I4/mmm* phase.

The enthalpy curves, *H*, were then calculated by interpolating the *V* – *p* and *E* – *V* curves with the third-order Birch–Murnagham equation^36,37^ to obtain the relation *H* = *E* + *pV*, where *E* is the total electronic energy of the system, *p* is the hydrostatic pressure to which the system is subjected to, and *V* is the volume per formula unit. The calculation of the relative enthalpy curves, with respect to the lowest enthalpy at 0 GPa of the *Cmc*2_1_ phase, allows the analysis of the evolution of the thermodynamic stability of the several structural phases, relative to one another, with increasing values of applied pressure (up to 20 GPa).

For the (harmonic) lattice-dynamics calculations, the supercell finite-displacement method was considered, with the Phonopy software package,^38^ where QE is used as the second-order force-constant calculator. The supercells employed to compute the phonon dispersion spectra were a 2 × 2 × 2 expansion of the primitive-cell. Phonon calculations were performed for the *Ccce* structure at several pressure values above which the system is energetically more favorable than the *Cmc*2_1_ phase. Respective calculations were considered in order to probe the dynamical stabilities of the *Ccce* phase. Phonon frequency calculations were also considered for the *Cmc*2_1_ system at pressure values between 20 and 30 GPa.

In the harmonic model, the equilibrium distance among atoms is not temperature dependent. The anharmonic effects needed to account for thermal expansion can be introduced by the QHA, in which the thermal expansion of the crystal lattice is obtained from the volume dependence of the phonon frequencies.^39,40^ To perform a QHA calculation, the phonon frequencies are computed for a range of expansions and compressions about the 0 K equilibrium volume, and the constant-volume free energy, for each configuration, is then evaluated as a function of temperature. From this approach, the equilibrium volume, bulk modulus, and Gibbs free energy can be obtained at several temperature values by fitting the free energy as a function of volume to the Vinet equation of state (EoS).^38,39,41^ We have performed QHA calculations for the two structural phases of interest: *Cmc*2_1_ and *Ccce*. The EoS fit was computed for a constant pressure of 19 GPa and for a temperature range up to 500 K (more details on the EoS fitting is discussed in the Supporting Information).

## 3 Results and Discussion

### 3.1 Sr_3_Hf_2_O_7_ Polymorphs

Since the environment around the Sr atoms is inequivalent, the general formula of the SHO system can be better written as (AHfO_3_)_2_A′O, where A′ = Sr_1_ and A = Sr_2_. Just as other RP structures, the system results from the inter-growth of rock-salt (R) and perovskite (P) blocks. The P blocks are composed of two layers of HfO_6_ octahedra along the *a*-axis, sharing the O corner ions. The A′-site cations occupy a ninefold coordination site (R block), while the A-site cations have a coordination number of 12 (cuboctahedral in P block).

The high-symmetry structure is centrosymmetric and belongs to the *I4/mmm* space group (S. G. n. 139).^17^ By decreasing the temperature, lower symmetry structural phases may be generated by inducing tiltings and/or rotations of the O octahedra cages. In ref (17) it was theoretically shown that second-order phase transitions may occur, toward lower structural phases, to the *Cmcm* or *Cmca* space groups, from which the phonon instability modes of these two phases may direct the final transition to *Cmc*2_1_ space group. Interestingly enough, it was experimentally observed that other related RP systems, such as Ca_3_Mn_2_O_7_ and Ca_3_Ti_2_O_7_ compounds, show a structural transition path from low-temperature *Cmc*2_1_, intermediate-temperature *Ccce* (S.G. n. 68), and high-temperature *I4/mmm*.^18^ Since the *Ccce* phase is not related by group-symmetry analysis to the *I4/mmm* → *Cmc*2_1_ transition and there is no single mode connecting the phases, we infer that this structural phase may occur as a first-order phase transition. Such a transition can occur by applying an external perturbation, such as pressure, in order to induce the condensation of the X_1_^–^ zone boundary mode from the centrosymmetric system.^17^

**Figure 1:**
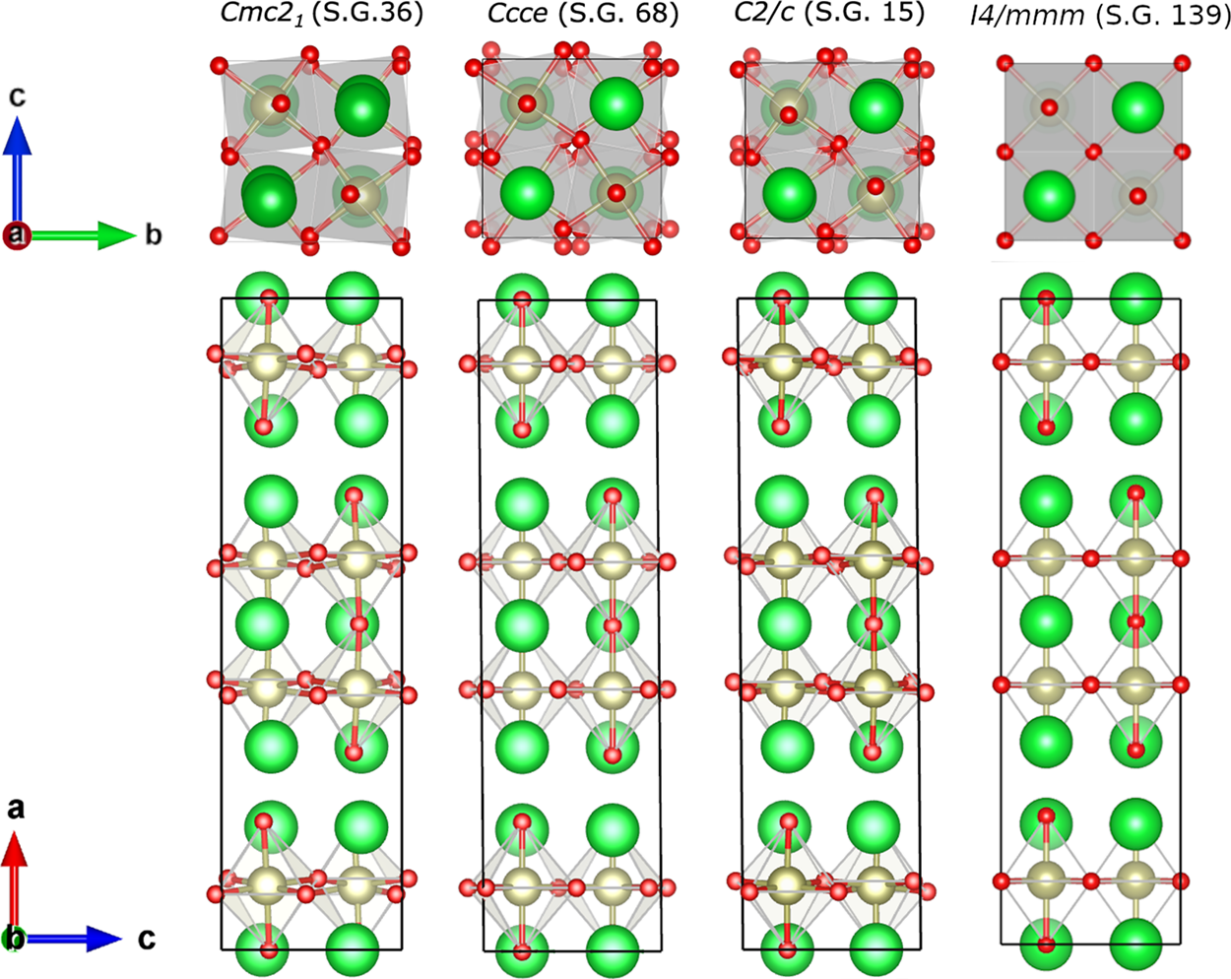
Representation of the *Cmc*2_1_, *Ccce*, *C2/c*, and *I4/mmm* structural phases of Sr_3_Hf_2_O_7_. The red, green, and gold spheres represent, respectively, the oxygen, strontium, and hafnium ions. The VESTA visualization software was used to plot the figures.^42^

We show in Figure 1 the four structural phases we have studied in this investigation. They are:

1. The ground-state structural phase at room conditions, evidencing a polar symmetry, *Cmc*2_1_ (S.G. n. 36). This system phase is related to the centrosymmetric *I4/mmm* structure through group-subgroup relations, by the condensation of the X_2_^+^ and the X_3_^–^ modes, which respectively lower the high-symmetry to the *Cmcm* and *Cmca* phases.^17^
2. The *Ccce* structural phase (S.G. n. 68), which has experimentally been observed in other similar RP compounds;^18^ however, such a phase has not been evidenced in SHO by analyzing the group-subgroup relations from the *I4/mmm* toward the polar *Cmc*2_1_ symmetry and as described in ref (17). Respective polymorph is induced through the condensation of the X_1_^–^ mode, which does not occur spontaneously for the SHO system. Such a distortion mode would have to be induced by applying an external perturbation (i.e., hydrostatic pressure) to break the symmetry from *I4/mmm* towards *Ccce*. We must note that there is no group–subgroup relation (by considering single mode analysis) between the *Ccce* phase and the *Cmc*2_1_ phase.^17^
3. The *C2/c* system (S.G. n. 15) was obtained by mapping out the anharmonic potential energy surfaces by following the eigenvectors associated with the soft (imaginary) phonon mode, localized at the Γ-point, of the *Ccce* structure (a more detailed description has been added in Supporting Information). From this analysis, it is possible to obtain a lower energy structure, corresponding to the minima of the potential energy surface.
4. And finally, the high-symmetry *I4/mmm* structural phase.

All three structural polymorphs are related to the high-symmetry, high-temperature, *I4/mmm* phase through tiltings and/or rotations of the O octahedra. When external perturbation is applied, symmetry-breaking occurs, thus lowering the energy of the system.

### 3.2 Energetic Stability

**Figure 2:**
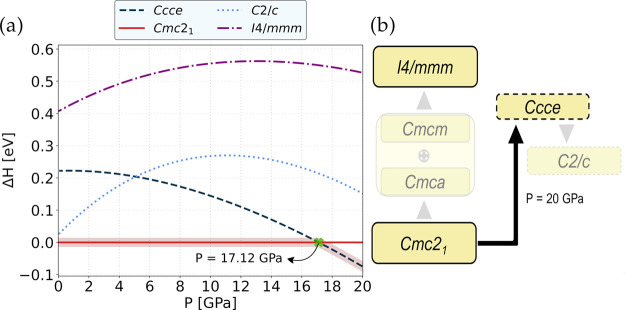
(a) Relative enthalpy curves of the *I4/mmm*, *Ccce*, *C2/c*, and *Cmc*2_1_ structural phases as a function of pressure with respect to the structure with the lowest energy value at ambient pressure (*Cmc*2_1_). The cross marker indicates the pressure value at which the *Ccce* structural phase becomes energetically stable. (b) Schematics of the most plausible transition pathways that Sr_3_Hf_2_O_7_ can undergo and based also on ref (17).

To probe whether the structural phases of Figure 1 can become energetically competitive as a function of hydrostatic pressure, the enthalpy energies, for pressures up to 20 GPa, have been calculated. These results are presented in Figure 2, where the relative enthalpy energies, Δ *H*, with respect to that of the structure with lowest enthalpy at 0 GPa (the *Cmc*2_1_ phase) are defined.

By analyzing Figure 2, we observe that at 0 GPa, the orthorhombic *Cmc*2_1_ phase is energetically the most stable as already discussed in the literature.^17^ The *C2/c* is slightly higher in energy than *Cmc*2_1_ (Δ *H* = ∼ 0.02 eV) at 0 GPa; however, it starts increasing the relative enthalpy as a function of pressure up until ∼10 GPa. After 12 GPa, the energetics of this system starts decreasing and, however, never becomes energetically stable throughout the studied pressure range. It is noteworthy of mentioning that the energetic tendency to decrease for increasing pressures evidences the possibility of the *C2/c* system stabilizing for pressures higher than 20 GPa. At 20 GPa, we observe a enthalpy difference of the *C2/c* system with respect to *Cmc*2_1_ of Δ *H* = 0.15 eV.

At room conditions, the *Ccce* phase is, in terms of enthalpy, the second highest system with respect to the ground-state structure. However, for increasing pressure, we observe a considerable decrease in energy. At ∼5 GPa, this system will be competing energetically with the *C2/c* structure, thus becoming more stable than the latter, and above 17.12 GPa, the *Ccce* phase becomes energetically the most favorable among the four studied systems.

The high-symmetry *I4/mmm* system shows quite elevated energy at 0 GPa, which is expected since it is a high-temperature phase. At around 10 GPa, we observe that the *I4/mmm* structural phase starts decreasing the enthalpy and, at 20 GPa, the energetic difference with respect to the *Cmc*2_1_ phase is around 0.52 eV. In comparison to the most stable phase at 20 GPa, the *Ccce* structure, the energy difference between the two phases is of ∼0.60 eV. Once again, we envisage the possibility of the high-symmetry *I4/mmm* system to stabilize at much higher pressures (>30 GPa), than those considered in the present work.

The enthalpy calculations were performed at 0 K, without taking into consideration the contributions to the free energy from the vibrations of the solids. In order to probe whether these effects could alter the energy ordering between the *Cmc*2_1_ and *Ccce* phases of Sr_3_Hf_2_O_7_, we have performed lattice-dynamics calculations on the equilibrium and compressed structures to evaluate the constant-volume Hemholtz (F) and constant-pressure Gibbs (G) free energies.

Within the HA, the Helmholtz free energy as a function of temperature can be obtained from the phonon frequencies and the lattice energy of the equilibrium structure. Temperature effects can be included through the Helmholtz free energy, which by introducing a transformation from the constant volume function, we obtain the thermal properties at constant pressure, and thus the Gibbs free energy.^40,43^ By increasing temperature, the volume dependence of the phonon free energy changes, which in turn results in different equilibrium volumes for different temperatures. This is regarded as thermal expansion in the QHA.

**Figure 3:**
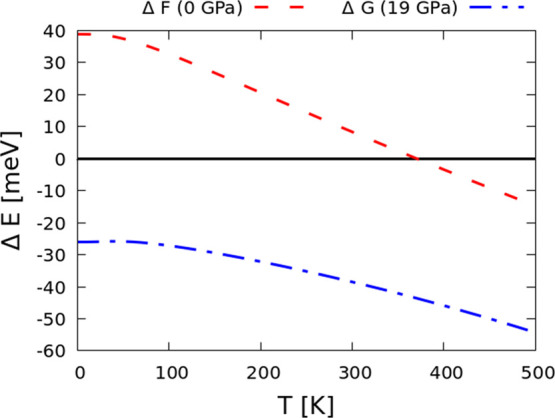
Harmonic (constant-volume/Helmoltz; red) and quasi-harmonic (constant-pressure at 19 GPa/Gibbs; blue) free energies of the *Ccce* phase relative to *Cmc*2_1_ (black) as a function of temperature.

The temperature dependence of the relative Helmholtz, Δ *F* (0 GPa), and Gibbs, Δ *G* (19 GPa), free energies of the *Cmc*2_1_ and *Ccce* phases are shown in Figure 3.

We observe from the Helmoltz free energy (Figure 3; red dashed line) that the *Ccce* structure becomes energetically more stable than the *Cmc*2_1_ system (Figure 3; black solid line), surpassing the latter at 370 K. This observation leads to the conclusion that the *Ccce* may also be induced through temperature effects, with the transition temperature being quite close to room temperature. At 0 K and 0 GPa, the free-energy difference between both phases is 38 meV, which is significantly lower than the relative enthalpy obtained without considering the entropy effects (∼220 meV).

Since the *Ccce* structure becomes dynamically stable around 19–20 GPa (more detailed information in Section 3.3) and that the *Cmc*2_1_ structure is still stable up until 30 GPa (Section 3.3), we have calculated the Gibbs free energy at 19 GPa (Figure 3; blue dashed-dotted line) to probe the evolution of the free energies of the two phases of interest. We observe that *Ccce* is thermodynamically the most stable phase throughout the studied temperature range (up to 500 K). At 0 K, the free energy of the *Ccce* phase differs from the *Cmc*2_1_ system with a relative value of Δ *E* ∼ 25 meV; as temperature increases, this energy difference also grows further apart (Δ *E* = 54 meV at 500 K). When comparing Δ *G* with Δ *H* (Figure 2) for the two systems at 0 K and 19 GPa, we observe that the enthalpy difference is ∼50 meV, which is in much closer agreement to when comparing the relative enthalpy with Δ *F* (for 0 GPa and 0 K).

We must note that the (quasi-)HA will not account for the influence of the soft modes of the *Ccce* system on the free energy, a deficiency which is amplified by the sensitivity of the volume to the free energy. Therefore, the transition temperature might be slightly underestimated than expected.^40^ Since at 19 GPa, neither the two analyzed phases evidence imaginary modes, we conclude that the QHA free energies would provide a much reasonable description of the free energy behavior between the two systems. Overall, and by comparing the free energies of Figure 3 to what was obtained for the relative enthalpy plots of Figure 2, we observe comparable behaviors between the energetics of both polymorphs: at 0 GPa and low temperatures, the *Cmc*2_1_ system is more stable than *Ccce*; whereas with pressure (19–20 GPa), the *Ccce* is more stable than *Cmc*2_1_, even for finite temperatures.

### 3.3 Dynamical Stability

Energetic stability is a necessary, but not a sufficient, condition for a structural phase to be synthetically accessible. Another condition that should be analyzed is the dynamical stability of the system, which requires the study of the phonon spectra. If imaginary frequencies emerge (usually represented by negative frequencies along the phonon dispersion curves), such a feature would indicate that the system is at a transient state, undergoing a phase transition and thus cannot be kinetically stable at the given temperature and/or pressure conditions.^44−49^

**Figure 4:**
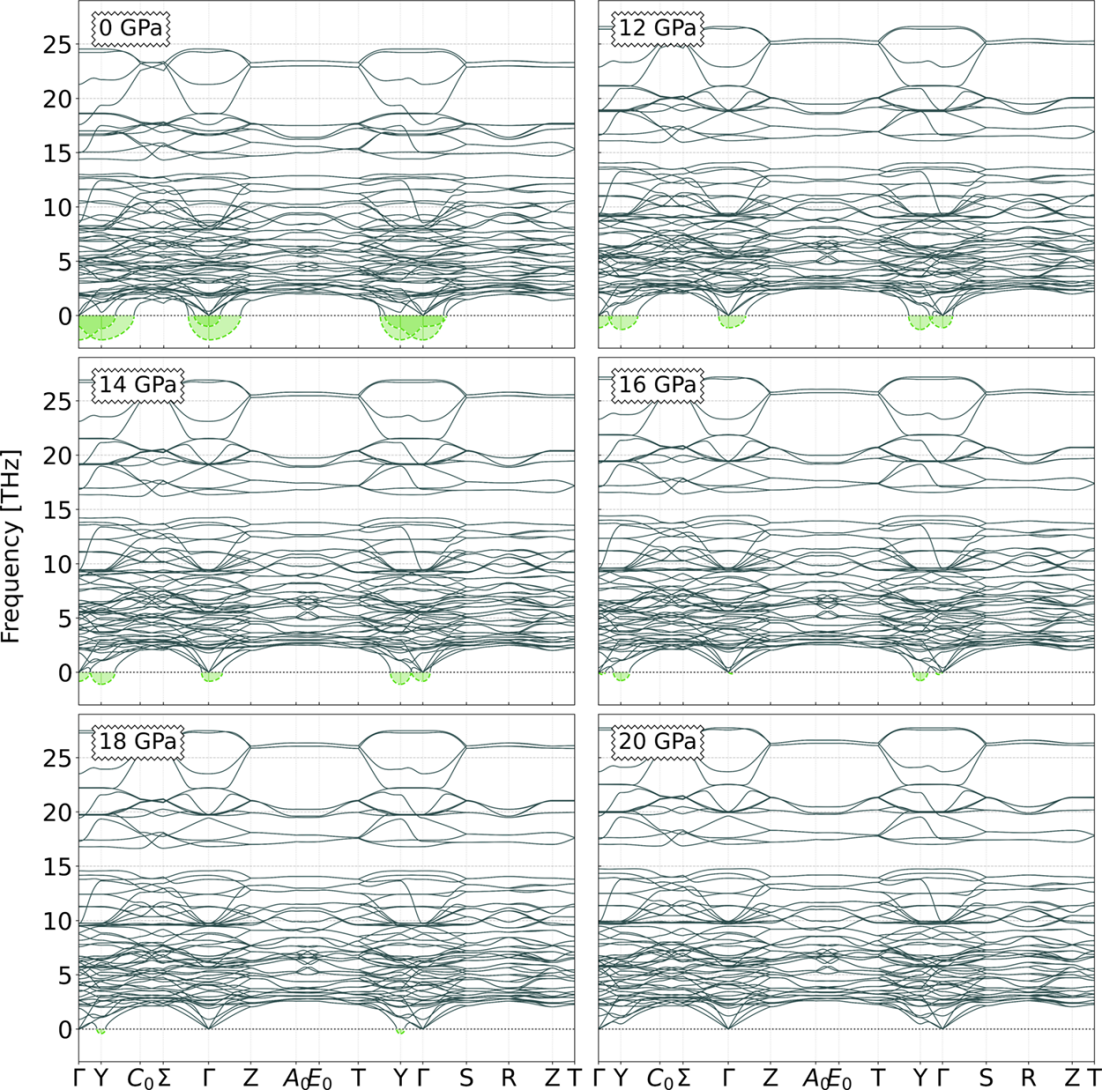
Phonon dispersion curves for the *Ccce* structural phase of the SHO system for several pressure values: 0 (taken from ref (17)), 12, 14, 16, 18, and 20 GPa.

Thus being, we have proceeded to calculating the phonon structure for the *Ccce* phase at several pressure values. These values were considered at: room pressure (and taken from ref (17) for comparison) and at values above which the energetical stability of the *Ccce* phase occurs – 12, 14, 16, 18, and 20 GPa, as shown in Figure 4.

As illustrated in Figure 4, the phonon dispersion curves of the *Ccce* structural phase at 0 GPa (retrieved from ref (17)) show negative phonon modes localized at two of the high-symmetry points of the BZ: Γ- and **Y**-points. We can therefore infer that such a structural phase is not kinetically stable at ambient pressure. However, we observe that as the applied pressure increases, the frequencies of the negative modes shift toward positive values. The Γ-point soft-mode hardens more rapidly than the **Y**-point: at 18 GPa, only a mild instability is observed at the latter point since at Γ, the soft optical mode already evidences positive frequencies. However, it is only at 20 GPa that we arrive at a structure that is both thermodynamically and dynamically stable, with the **Y** phonon mode evidencing positive values as well.

**Figure 5:**
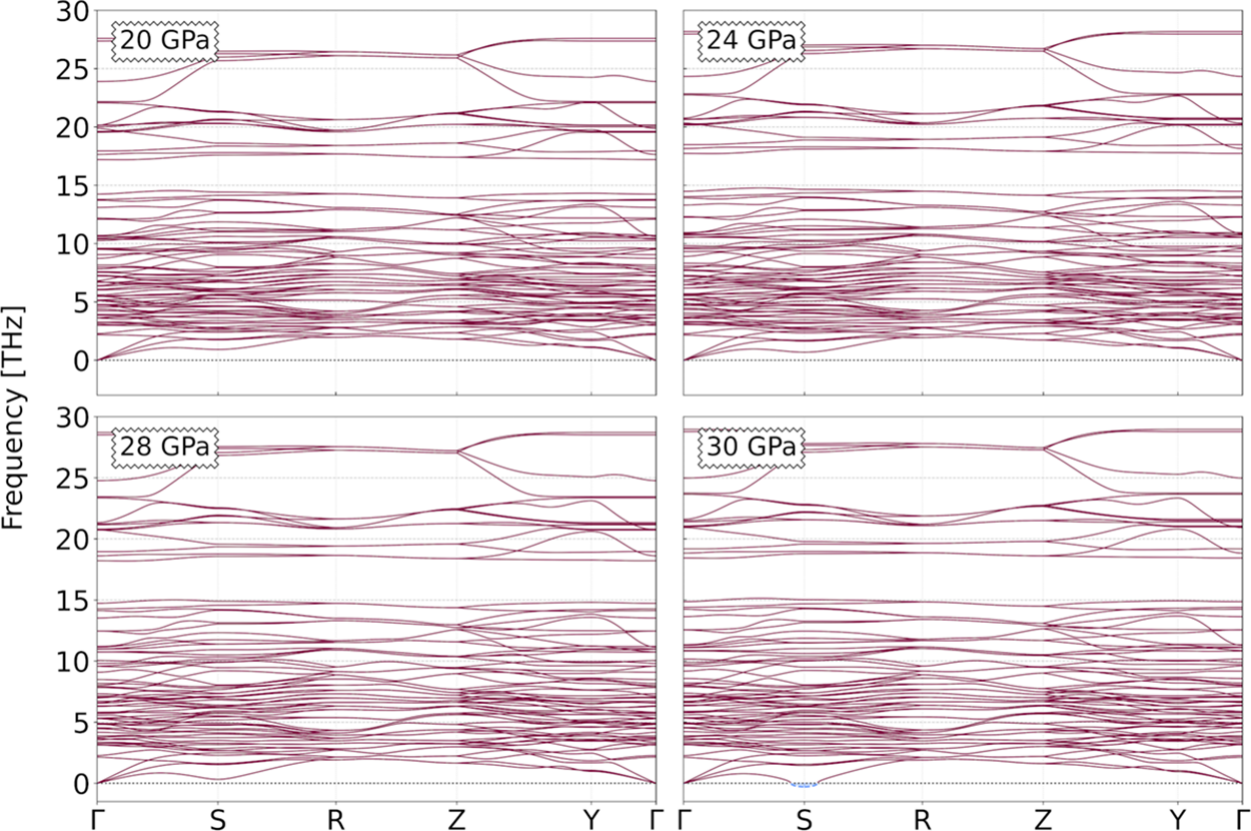
Phonon dispersion curves for the *Cmc*2_1_ structural phase of the SHO system for high pressures: 20, 24, 28, and 30 GPa.

We also show, in Figure 5, the phonon dispersion curves of the *Cmc*2_1_ system at several pressure values, and we observe that the structure is dynamically stable still at 20 GPa, thus coexisting with *Ccce* phase, as a metastable compound. It is noteworthy of mentioning that around the high-symmetry **S**-point, the lowest frequency branch starts to decrease toward a soft mode, which may be an indication that for higher pressure values, a structural transition will occur due to the instability at this **q**-point. Indeed, by increasing the pressure to 30 GPa, it is observed that this soft-mode becomes unstable, thus rendering this structure kinetically unviable above this pressure value.

## 4 Conclusions

In the present work, we have performed ab-initio calculations to study the thermodynamical stability of the room temperature *Cmc*2_1_ phase and the high-temperature *I4/mmm* structure; as well as different structural phases of Sr_3_Hf_2_O_7_, which are not related to the aristotype polymorph through group–subgroup relations, i.e., are a result of first-order transitions. The latter were the *Ccce* and *C2/c* structural phases.

It was observed that, at room conditions, the *Cmc*2_1_ phase is the most energetically stable, as expected; the *C2/c* phase being 0.05 eV higher in energy than the ground-state; followed by the *Ccce* system with an energy difference of ∼0.18 eV with respect to *Cmc*2_1_; and finally, the *I4/mmm* polymorph with the highest energy difference of ∼0.41 eV in relation to the ground-state phase. We observe that the enthalpy of *Ccce* decreases as pressure increases, and at ∼17 GPa, it surpasses that of the *Cmc*2_1_ structure, becoming energetically the most stable system of all four studied compounds.

To inspect in detail the two lowest energetic compounds, namely the *Ccce* and *Cmc*2_1_ phases, we have calculated the free energies (where entropy is accounted for) and the phonon dispersion curves to analyze their respective dynamical stability. From the constant-volume free energies, we observe that the *Cmc*2_1_ system is energetically the most stable compound below 370 K. Above this temperature, the *Ccce* polymorph has its free energy lowered, thus becoming more stable than *Cmc*2_1_ phase. By probing the volume-temperature dependent free energies at 19 GPa, we observe that the *Ccce* is always the most stable phase when compared to *Cmc*2_1_, throughout the studied temperature range, up to 500 K. When observing the phonon dispersion spectra, we conclude that the *Ccce* structure only becomes dynamically stable at 20 GPa, which is at a higher pressure range than when the energetic transition occurs from *Cmc*2_1_. The *Cmc*2_1_ phase, on the other hand, is dynamically stable up to 30 GPa when an imaginary mode at the high-symmetry **S**-point emerges. These results suggest that both phases may coexist energetically at pressure values from ∼19 GPa up to ∼30 GPa; at this pressure interval, the polar *Cmc*2_1_ structure is a metastable system.

From the present calculations, we conclude that the quasi-2D layered RP perovskite Sr_3_Hf_2_O_7_ compound has a stable phase at room conditions which is a ferroelectric ground-state system with *Cmc*2_1_ symmetry. By applying hydrostatic pressure, this phase may undergo a transition to the *Ccce* structure, for it becomes thermodynamically (at ∼17 GPa) and dynamically stable (at 20 GPa).

## Supporting Information Available

Evolution of the lattice parameters of the four studied structural phases with applied pressure, potential-energy surface for the phonon instabilities associated with the high-symmetry Γ-point negative mode of the *Ccce* phase, band structure of the *Ccce* and *Cmc*2_1_ structural phases, electronic band structure of *Cmc*2_1_ with spin-orbit coupling effects, and QHA: Helmoltz free-energy EoS fits, temperature-dependent volume, and the volumetric thermal-expansion coefficient (ZIP)
