# Supplementary figures and images for: Pressure-Induced Phase Transformations of Quasi-2D Sr3Hf2O7
Source: J Phys Chem C Nanomater Interfaces. 2023 Aug 1;127(31):15435–42. doi: 10.1021/acs.jpcc.3c01596 (PMC10497066; doi:10.1021/acs.jpcc.3c01596)

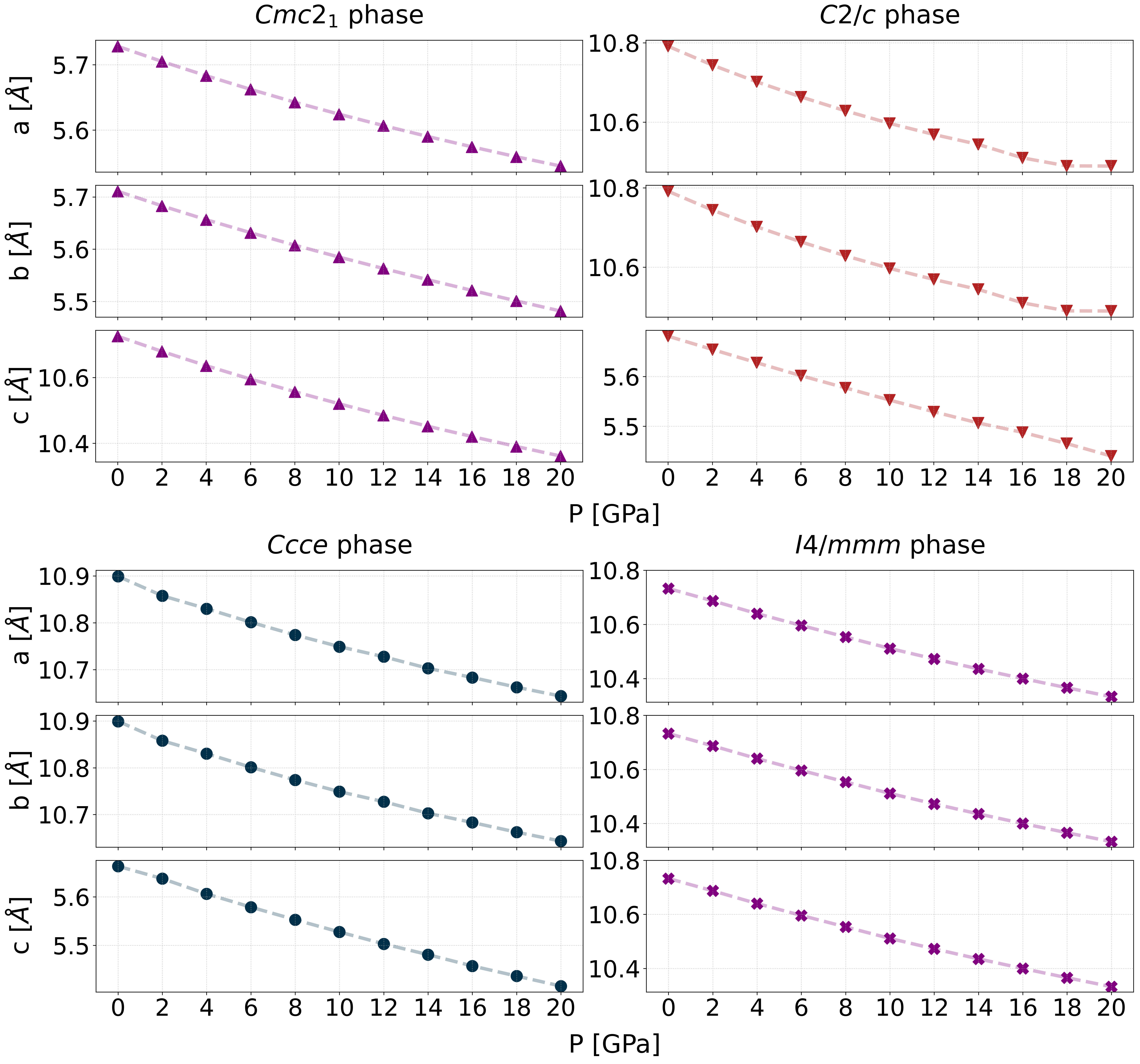

Supplement: Supplementary file 1 — jp3c01596_si_001.zip [file jp3c01596_si_001.zip › FigS1.png]

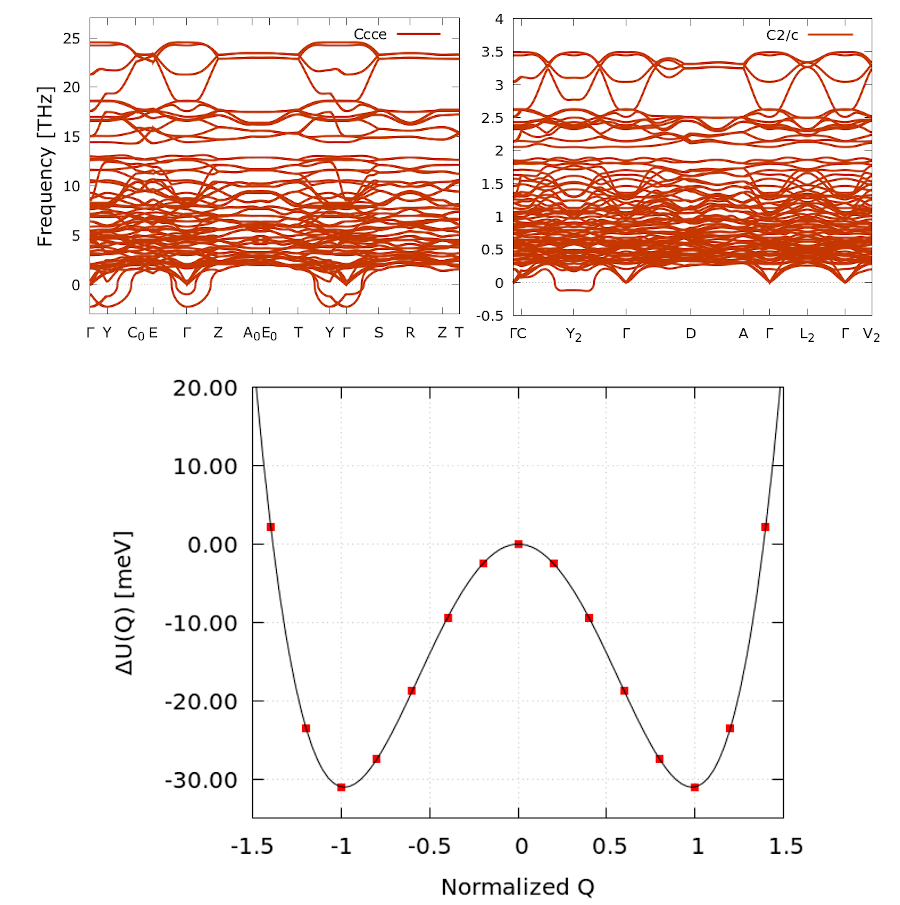

Supplement: Supplementary file 1 — jp3c01596_si_001.zip [file jp3c01596_si_001.zip › FigS2.png]

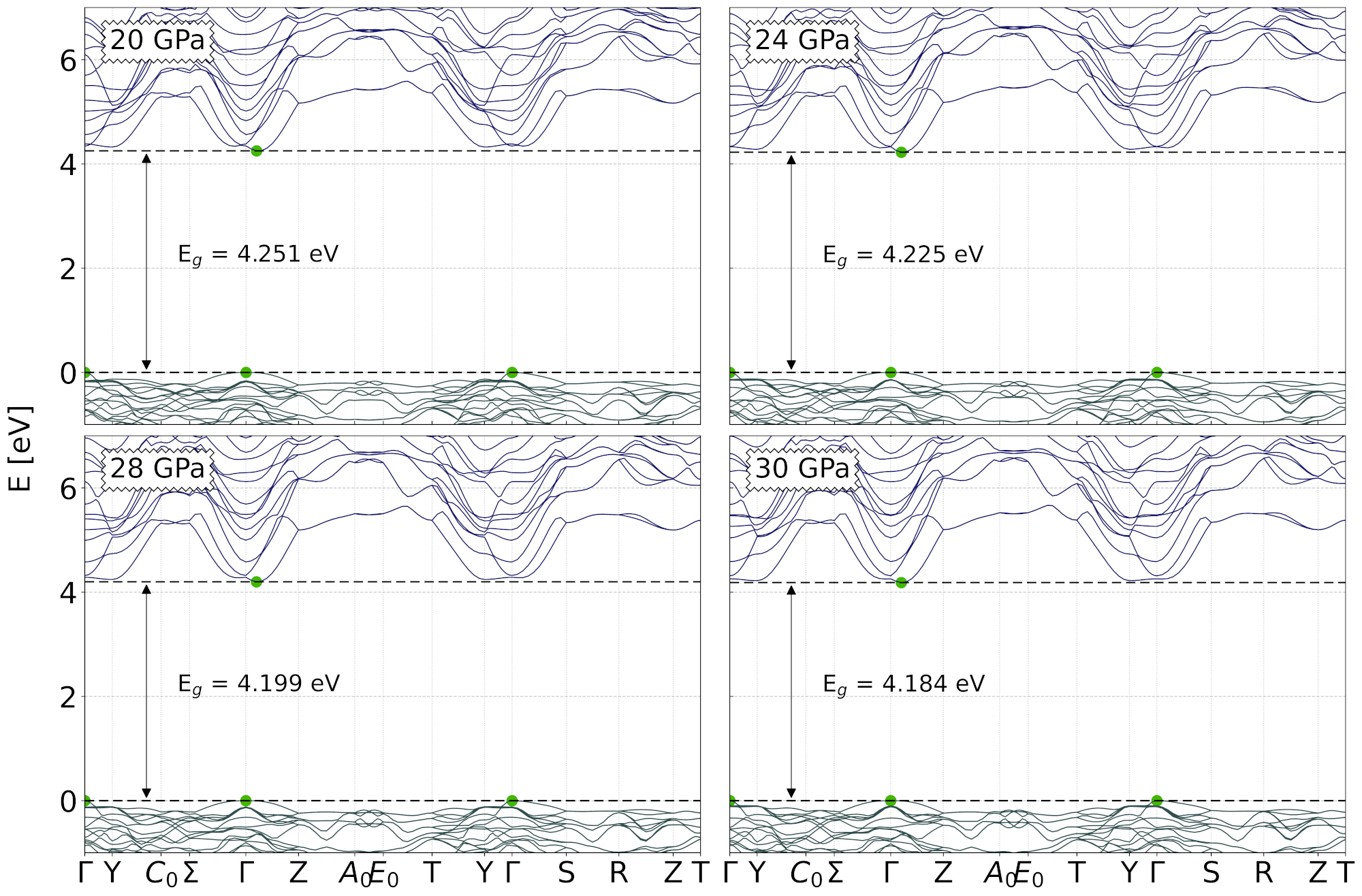

Supplement: Supplementary file 1 — jp3c01596_si_001.zip [file jp3c01596_si_001.zip › FigS3.png]

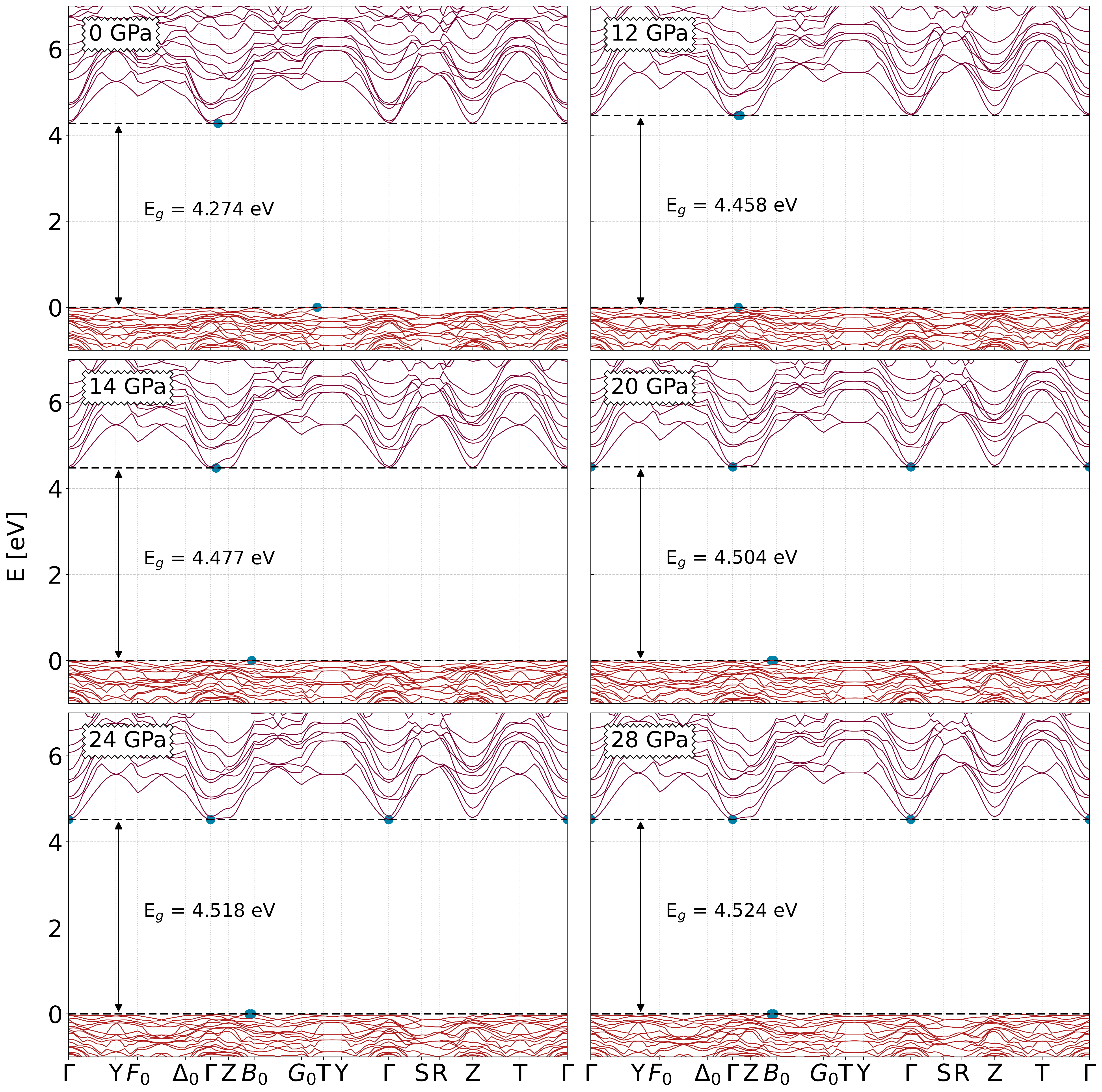

Supplement: Supplementary file 1 — jp3c01596_si_001.zip [file jp3c01596_si_001.zip › FigS4.png]

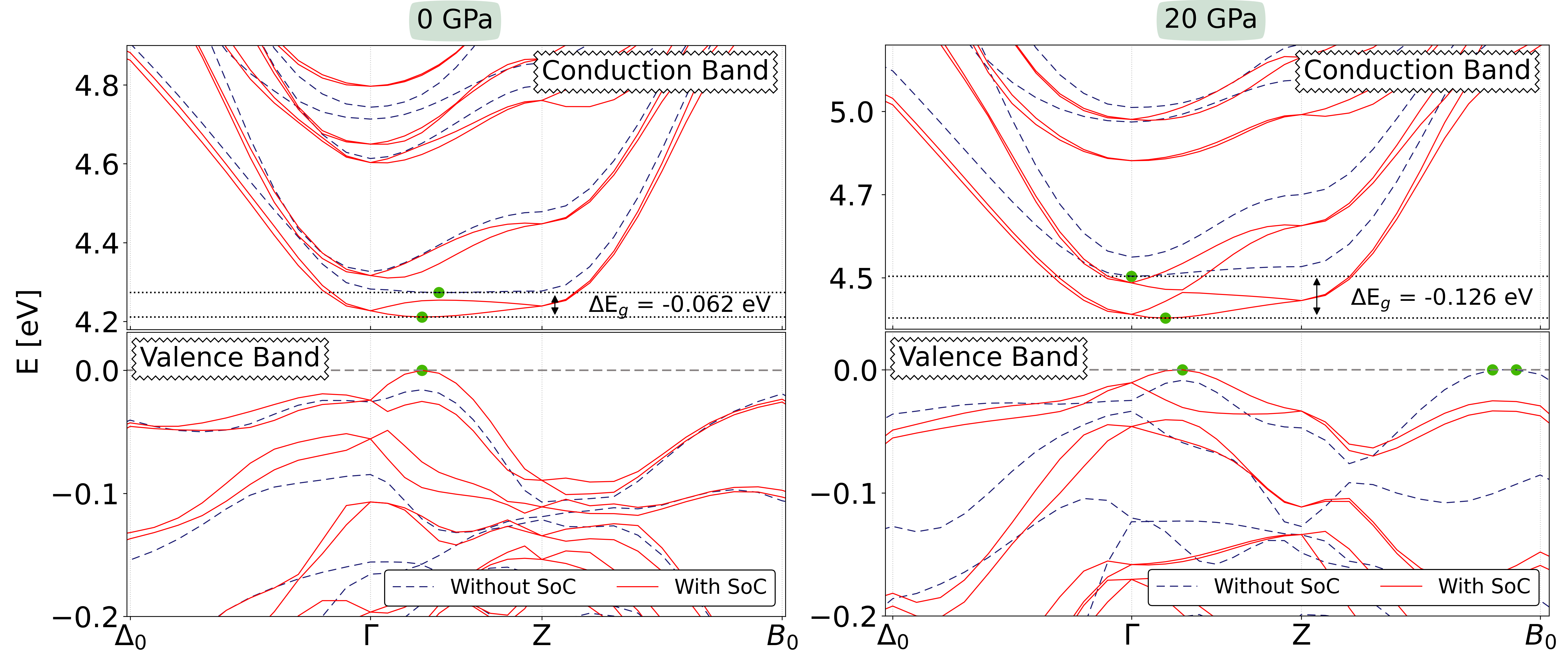

Supplement: Supplementary file 1 — jp3c01596_si_001.zip [file jp3c01596_si_001.zip › FigS5.png]

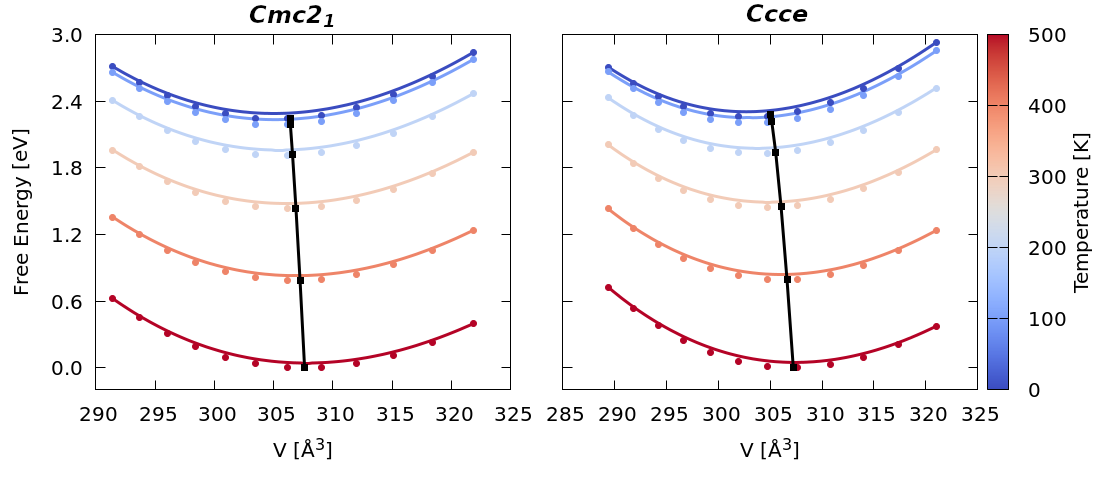

Supplement: Supplementary file 1 — jp3c01596_si_001.zip [file jp3c01596_si_001.zip › FigS6.png]

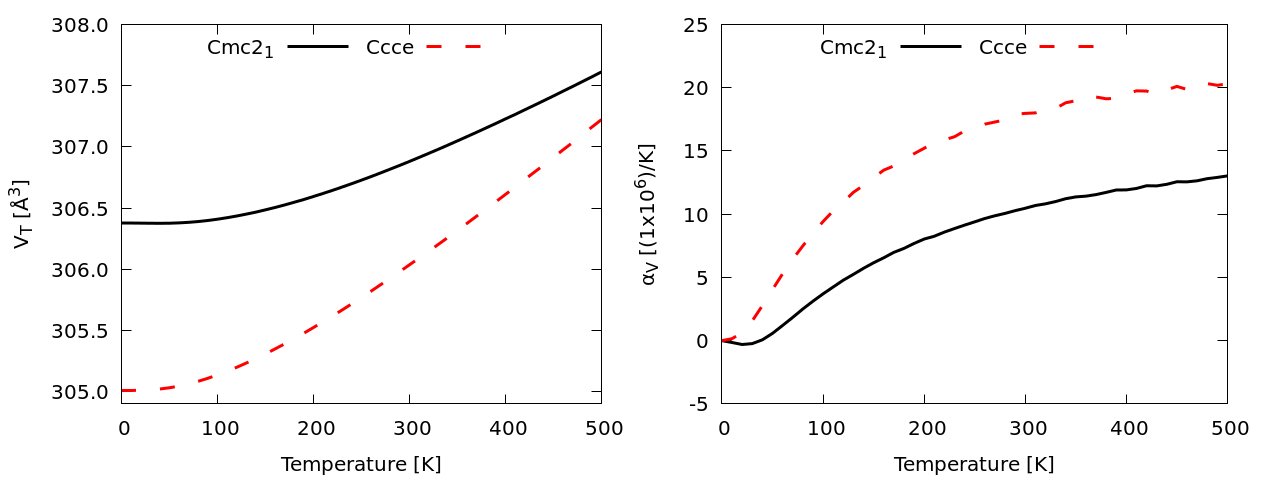

Supplement: Supplementary file 1 — jp3c01596_si_001.zip [file jp3c01596_si_001.zip › FigS7.png]
